# *Porphyromonas gingivalis* Periodontal Infection and Its Putative Links with Alzheimer's Disease

**DOI:** 10.1155/2015/137357

**Published:** 2015-04-30

**Authors:** Sim K. Singhrao, Alice Harding, Sophie Poole, Lakshmyya Kesavalu, StJohn Crean

**Affiliations:** ^1^Oral & Dental Sciences Research Group, School of Medicine and Dentistry, University of Central Lancashire, Preston PR1 2HE, UK; ^2^Department of Periodontology, College of Dentistry, University of Florida, Gainesville, FL, USA

## Abstract

Periodontal disease (PD) and Alzheimer's disease (AD) are inflammatory conditions affecting the global adult population. In the pathogenesis of PD, subgingival complex bacterial biofilm induces inflammation that leads to connective tissue degradation and alveolar bone resorption around the teeth. In health, junctional epithelium seals the gingiva to the tooth enamel, thus preventing bacteria from entering the gingivae. Chronic PD involves major pathogens (*Porphyromonas gingivalis*, *Treponema denticola*, and *Tannerella forsythia*) which have an immune armoury that can circumvent host's immune surveillance to create and maintain an inflammatory mediator rich and toxic environment to grow and survive. The neurodegenerative condition, AD, is characterised by poor memory and specific hallmark proteins; periodontal pathogens are increasingly being linked with this dementing condition. It is therefore becoming important to understand associations of periodontitis with relevance to late-onset AD. The aim of this review is to discuss the relevance of finding the keystone periodontal pathogen *P. gingivalis* in AD brains and its plausible contribution to the aetiological hypothesis of this dementing condition.

## 1. Introduction

*Organ Specific Inflammation*

*(a) The Oral Cavity*. Bacterial diseases such as chronic periodontal disease evoke the host's immune responses, involving both the innate and adaptive signalling mechanisms with cell recruitment from the systemic circulation (macrophages, plasma cells, and T and B lymphocytes) that infiltrate the gingival soft tissues [1]. In periodontal disease (PD) there is paucity of information concerning the molecular pathways in the host cells, which could regulate their* inflammatory* response to complex subgingival bacterial biofilms. In the gingival inflammatory infiltrate, the outcome of the cytokine/chemokine response is regulated by the production of proinflammatory cytokines that are encountered by anti-inflammatory cytokines [2].* Porphyromonas gingivalis* (*P. gingivalis*) has been proposed as the keystone periodontal pathogen in maintaining the PD associated inflammophilic microbiota [3, 4]. An inflammatory pathology is a disease condition where the host's immune defences constantly encounter invading pathogens or bacterial virulence factors. The host's defence failure in the gingival tissues, leads to the keystone inflammophilic microbe,* P. gingivalis*, colonising the subgingival areas and subsequently disseminating to distant organs [5–7]. The subversive armoury of this bacterium not only provides nutritional sustenance from sustainable inflammatory milieu but also allows for continued survival of* P. gingivalis *in the highly toxic niches [8].

*(b) The Brain*. The brain is immunologically privileged due to a physical blood brain barrier (BBB) and due to the absence of a lymphatic system and therefore displays low levels of molecules that are critical to antigen presentation. Thus, during neurodegeneration, the brain relies less on the recruitment of peripheral adaptive immune surveillance cells but more on the resident central nervous system (CNS) cells to recognise and mount a response against the invading pathogens. Although astrocytes and neurons have the capacity to deal with infection, it is the microglial cells that serve as the primary protective guardians of the healthy brain. Susceptibility to microbial infection appears to increase during advancing age and just before clinical diagnosis of dementia is confirmed [9]. This is attributed to the fact that, during aging, microglia appear to develop functional defects [10]. Thus, it can be argued that where age is a known risk factor (as in AD), infections will play a major role in the specific disease processes leading to dementia.

## 2. The Oral Cavity

The oral cavity is equally important for both nutritional and communication functions which are necessary for a healthy and social existence in animal species as well as for bacteria which exist in this niche. It includes a variety of specialised structures including teeth, keratinised and non-keratinised mucosa, gingivae, periodontal structures, salivary glands, and specialist linings for taste. The oral cavity has its own protective barriers to reduce colonisation of these bacteria including the buccal, gingival, and tongue mucosal epithelial surfaces [11]. These surfaces work together with the internal secretions such as saliva, mucous, and the gingival crevicular fluid to protect the epithelial membrane [12]. Saliva contains a range of both innate and adaptive immune molecules designed to minimise the attachment and survival of organisms that may establish within the gingival surface [13]. Chemical factors such as antimicrobial peptides including defensins (*α*-defensins expressed in neutrophils; *β*-defensins from gingival mucosa) [14–16] are an example of the innate immune mechanisms, which are involved in controlling pathogenic bacterial colonisation. Measures controlling the adaptive immune response include immunoglobulins (IgA) specific for the mucosal surfaces [17, 18] and various enzymes (lactoferrin and lysozyme) designed to prevent bacterial metabolic processes [19] essential for their colonisation and lysis.

In addition, the gingival mucosa has developed a direct protective response against bacterial antigenic challenges through the action of cell-mediated immunity within the periodontal structures [20, 21] via antigen presentation. This results in the subsequent infiltration of T and B cells, a hallmark of the host's response to the presence of these non host organisms [20–23]. If the balance between natural protective barriers and bacterial colonisation is disrupted, the bacterial load increases in favour of oral inflammatory conditions such as PD [24] in susceptible individuals.

## 3. Periodontal Disease

There are two distinct inflammatory conditions that make up PD: gingivitis, a plaque-induced, reversible condition; and chronic periodontitis, an irreversible condition.

### 3.1. Gingivitis

Gingivitis represents the most prevalent disease to affect the periodontium, affecting up to 60% of teenagers and 40–50% of adults [25]. Gingivitis is caused by the accumulation of dental plaque and may manifest after only days or weeks following plaque accumulation [26], whereas periodontitis takes longer to develop [27]. To support the aetiological link between plaque and gingivitis, studies aiming to reduce or eliminate dental plaque have led to disease resolution [26, 28, 29]. Differences in susceptibility of individuals to gingivitis have been demonstrated. It has been suggested that these differences have been down to the microbial makeup of the plaque or the rate of plaque accumulation [26]. Local factors that may encourage an increased plaque accumulation, and therefore increase the susceptibility to gingivitis, include developmental or anatomical tooth variations, pathological tooth conditions, caries, frenum position, gingival developmental conditions, and iatrogenic factors (e.g., overhangs on restorations) [28]. Systemic factors may also contribute to an individual's susceptibility to gingivitis. These include metabolic factors (e.g., puberty, pregnancy), genetic factors, environmental factors (e.g., smoking, medication, and nutritional status), and systemic conditions (neutropenia, HIV disease) [28]. More recently, the individual's susceptibility (irrespective of quantitative or qualitative plaque accumulations) to the development of gingivitis has been investigated. Trombelli et al. [29] experimentally demonstrated two distinct groups, “high responders” and “low responders.” These groups showed significantly different severities of gingivitis to similar plaque accumulation rates. These results reinforce the hypothesis of subject-specific effects of poor oral hygiene on gingival tissues. Although gingivitis does not always progress to periodontitis, it has been shown that individuals who show an increased susceptibility to gingivitis will also have an increased susceptibility to developing periodontitis, although, to date, there are no reliable means to predict susceptibility to periodontitis [30].

### 3.2. Periodontitis

Periodontitis is the most common infectious inflammatory disease of humans. A recent survey in the USA concluded that 47.2% of adults aged 30 years and older had periodontitis [31]. Periodontitis is a chronic inflammatory destruction of the gingival connective tissue attachment to the root surface, cementum, and adjacent alveolar bone resulting from continuous inflammatory response triggered by subgingival bacterial biofilm. The causal bacteria typically include* P. gingivalis*,* Treponema denticola* (*T. denticola*), and* Tannerella forsythia* (*T. forsythia*) [24]. The established PD lesion is the subgingival pocket, which has variable (5–10 mm) depth, in which 1 × 10^9^ mixed communities of inflammophilic bacteria can flourish around the teeth [12]. These increased numbers represent a significant bacterial load with the loss of collagen fibers and attachment to the cementum surface, apical migration of junctional epithelium, formation of deep periodontal pockets, and resorption of alveolar bone. Alveolar bone is continuously undergoing the process of remodelling such as constant bone resorption by osteoclasts followed by a phase of bone formation by osteoblasts through a coupling mechanism.

## 4. Inflammatory Bacterial Challenge Driving Periodontal Disease Process

In PD, the inflammatory response is initiated by bacteria but also involves other molecules known as pathogen associated molecular patterns (PAMPs). These include lipopolysaccharide (LPS), capsular proteins, flagellin, fimbrillin, peptidoglycan, bacterial DNA, proteases, (gingipains, dentisilin, and trypsin-like proteases) [32], and other protein modifying enzymes [33], which act to both stimulate and/or dampen the hosts' innate immune responses [1, 33]. The stimulation causes hosts' pattern recognition receptors (PRRs) to produce a range of cytokines which recruit appropriate immune cells to the site of infection [32]. In addition, the metabolic products of bacteria cause the periodontal pocket epithelium to secrete neuropeptides (such as substance P, calcitonin gene-related peptide, and vasoactive intestinal polypeptide) that promote vasodilation of local blood vessels and permit an influx of neutrophils in response to signals from chemokines [1]. The immune dampening response accompanying the bacterial infection protects the periodontopathogen (*P. gingivalis*) by compromising the host's innate defences in diverse ways [33–35].

## 5. *P. gingivalis*, the Keystone Pathogen, and Its Ability to Bypass Inflammatory Signalling Cascades

A keystone pathogen is defined as an organism that can hold an entire arch together [3, 36]. Holding the arch can be viewed as an essential scaffold that acts as a guardian for the survival of new inflammophilic synergistic microbial communities in the same ecological niche. In PD, this crucial bacterium is said to be* P. gingivalis*. The metabolic dominance of this bacterium in terms of its population in PD appears to be maintained at low levels, perhaps to avoid direct competition within the same species or for natural selection of a dominant bacterium. Its mastery at evading hosts' immune mechanisms to evoke and sustain inflammatory milieu via a number of signalling cascades affords protection not only to itself but also to other inflammophilic organisms that may be less able to survive such toxic conditions [8]. The main innate immune system signalling pathways for bacteria and/or their products include the integrin receptor CR3 (CD11b/CD18) and toll-like receptor (TLR) signalling [37] and the complement cascade [38]. For a fuller appreciation of the complement cascade involvement [38] in PD, see Hajishengallis et al. [4] and Hajishengalis [37]. In brief, a functioning complement system comprises of at least three different activation pathways (classical, alternative, and the mannan binding lectin), all of which converge upon the central component C3 which then leads to the terminal pathway activation. Through this activation process, numerous enzymatic activation fragments are generated many of which have immunomodulatory functions; examples of these include the anaphylatoxins C3a, C5a and the cytolytic membrane attack complex (MAC). There is cross talk between these pathways for cytokine liberation, and other downstream signalling pathways such as the extracellular signal-regulated pathways of activation which are also fully described by Hajishengalis [37].

## 6. Virulence Factors of* P. gingivalis*

Holt et al. [39] extensively reviewed the macromolecules associated with* P. gingivalis* that,* in vivo*, might function in the inflammatory and destructive events of PD. These include the capsule, outer membrane, its associated LPS, fimbriae, proteinases, and selected enzymes. LPS is an integral component of all bacteria and is found in the outer membrane layer. LPS may also be found in a cell-free form occurring after bacterial autolysis as a result of exposure to antibiotics during rapid growth or when essential nutrients have depleted from the environment. It is a stable molecule, which can withstand heating to 100°C for several hours. When in the host, main role of LPS appears to be linked to priming of tissue specific antigen presenting cells [40].

*P. gingivalis *LPS is generally of smooth type [41], consisting of three regions: lipid A, R polysaccharide, and O polysaccharide [42]. The lipid A region of LPS in many cell types promotes antigen presenting cell (neutrophils, dendritic cells) and activation and secretion of proinflammatory cytokines and nitric oxide [43]. This can result in the stimulation of prostaglandin and leukotriene production and activation of the complement cascade and the coagulation cascade [44]. Hence, high levels of LPS within the host can result in an elevated immune response, which, once above the hosts' threshold level, will result in damage to the tissue [44].* P. gingivalis* contains two LPS macromolecules, an O-LPS containing the O polysaccharide attached to the lipid A core and an A-LPS where the phosphorylated branched, mannan repeating unit is also attached to the lipid A core [45]. In addition,* P. gingivalis *LPS possesses significant amounts of lipid A heterogeneity containing tetra- and penta-acylated structures. The heterogeneity of LPS includes differences in the number of phosphate groups together with both the amount of lipid A fatty acids and their specific position. The presence of multiple lipid A structures makes it more difficult for the innate host responses to recognise the molecule thereby aiding the virulence of* P. gingivalis* [34].

Another important factor regarding the LPS of* P. gingivalis *is its ability to modify certain surface proteins. Veith et al. [35] demonstrated that the gingipain protease complex from* P. gingivalis* W50 has an intimate association with LPS glycosylation sites of these proteins. These glycosylation sites give rise to the cross-reactivity between monoclonal antibodies against LPS and to the carbohydrate moieties on gingipains [35]. O'Brien-Simpson et al. [46] suggested that the modification recognised by the* P. gingivalis* monoclonal antibody (clone 1B5) is located in the C-terminal segment of RgpB, suggesting that gingipains can be modified by LPS attachment to the conserved C-terminal segment. This could have implications in the virulence of this pathogen as it displays a mechanism by which the gingipains attach to the outer membrane [47]. LPS also activates the immune response through PRRs on the membrane of host cells or with both the tetra- and penta-acylated lipid A structures of* P. gingivalis*. The latter differentially activate the TLR-mediated nuclear factor kappa enhancer of B cell (NF-*κ*B) signalling pathway.* P. gingivalis *LPS has been shown to use both TLR-2 and TLR-4, depending on the cell type [48]. Bacteria also secrete exotoxins, proteins (often enzymes and metabolic by-products) that exert damage on the host following their release. These PAMPs include proteases, coagulases, and fibrinolysins, which act on their specific substrates [39, 47, 49]. For example,* P. gingivalis *has the ability to secrete a large variety of enzymes such as peptidylarginine deiminase, an enzyme which can modify free or peptide-bound arginine to citrulline [33, 50]. Others include collagenases, which break the peptide bonds in collagen (the main structural protein of connective tissues) [51].

*P. gingivalis* is also armed with two types of gingipains, lysine specific (Kgp) and arginine specific (Rgps) as determined by the specificity for their cleaving sites [52]. Gingipains are known to play a major role in the progression of PD, inducing inflammation and tissue destruction in the periodontium [53].

Peptidoglycan is the only cell wall component common to all Gram-negative and Gram-positive bacteria and is the essential scaffold of all cell walls that provides rigidity. Peptidoglycan, the major component of Gram-positive bacterial cells, is formed of glycan strands cross-linked via short peptides. The glycan segment comprises two alternating amino-hexose sugars: N-acetylglucosamine and N-acetyl muramic acid, cross-linked by short chains of amino acids. Usually, L-alanine is bound to muramic acid in Gram-positive bacteria, or mesodiaminopimelic acid in Gram-negative bacteria [54–56]. Peptidoglycan is capable of inciting innate immune responses in general [57, 58] and to those that ultimately contribute to sepsis [59]. Both bacterial peptidoglycan and its products (muramyl peptides) have been shown to act as inflammatory mediators by activating host's innate PRR (TLRs) and intracellular signalling receptors such as nucleotide-binding oligomerization domain or NOD 1 and NOD 2 [60]. Of these, TLR-2 is one that recognises peptidoglycan through MyD-88/NF-*κ*B subsequently leading to chemokine/cytokine release [57, 58].

A genome-wide* in vitro* expression profile of neutrophils [40] demonstrated that the cord blood following challenge by the Gram-positive bacterial peptidoglycan was capable of activating neutrophils. Neutrophil activation was detected by expression of the integrin receptor CR3 (CD11b) and the NF-*κ*B signalling pathway of cyto/chemokine release (TNF-*α*, IL-8) [40]. During the process, significant production of reactive oxygen species (ROS) was also recorded, suggesting that peptidoglycan may be a potential source of ROS during an infectious episode [40].

## 7. Immune Evasion Strategies of* P. gingivalis*

Plethora of data supports the idea that* P. gingivalis* is a master evader of the host's immune system [33–35, 61–67]. The structural nature of a biofilm provides a physical barrier against immune cells of the host [68]. In addition, a number of active mechanisms are employed by bacteria including degradation of complement fragments (avoiding opsonisation by protease digestion of complement fragments), recruitment of hosts' regulatory proteins (Factor H, C4 binding protein), and protection by the bacterial cell wall. In the latter, either the MAC is unable to form, or their cell wall component (polysaccharides) mediated complement activation is suppressed [63, 69–71].* P. gingivalis* is very resistant to destruction by complement due to the ability of the gingipains to degrade C3 and C5 thereby preventing the deposition of C3b on the surface of the bacterial cell walls [67, 72]. Gingipains have been found to be modified by, and reactive with, an LPS recognising monoclonal antibody (MAb 1B5) suggesting a mechanism for the attachment of the RgpA and Kgp complexes to the outer membrane [35]. This attachment potentially increases the virulence of the bacterium, as gingipains present on the surface of the bacterial cell wall will be readily available to degrade complement proteins thus evading the hosts' immune response. Gingipains can also attach to C4b binding protein avoiding destruction by complement mediated lysis [63].

Another immune evasion mechanism demonstrated by* P. gingivalis *is its adherence to erythrocytes via complement receptor 1 (CR1). This enables the bacteria to pass undetected by circulating phagocytes and provide a potential transport mechanism for the movement of* P. gingivalis *via the systemic circulation [66]. The additional ability of* P. gingivalis* to alter the lipid A structure of LPS could be one of the strategies utilised to evade innate host defence in gingival tissues potentially contributing to the pathogenesis of PD [73]. Our in-house data has demonstrated cleavage of the CD14 receptor from the neuroblastoma (IMR32) cell line during* in vitro* exposure to* P. gingivalis *crude culture supernatant containing abundant gingipains.

*P. gingivalis* strains are also capable of evading the innate immune recognition by degrading complement component C3 [61] and can intercept the cross talk at the C5 convertases stage by expressing peptidylarginine deiminase enzyme to inactivate C5a for its own survival [33].

The immune invasion strategies of* P. gingivalis *are of great importance not only in PD, but also in relation to systemic disease as this bacterium and its virulence factors access the systemic organs. In doing so,* P. gingivalis *and/or its products and any inflammatory mediators generated within the blood can potentially reach remote body organs. Routine dental procedures, including dental extraction, periodontal surgery, tooth scaling, and even tooth brushing and flossing seed oral bacteria into the systemic circulation [74–77]. Fitting with the theory of “focal infection” periodontal bacteria have the potential to go undetected by the immune system (via immune evasion mechanisms) and ultimately access remote body organs of individuals with susceptibilities for developing inflammatory pathologies. Currently, PD has been linked directly with cardiovascular disease [78–80], diabetes mellitus [81], respiratory infections [82, 83], rheumatoid arthritis [84, 85], osteoporosis [86], obesity [87], and adverse pregnancy outcomes [88] including low birth weight [89] and preterm birth [90]. Most recently PD has been linked with the aetiology of AD [91–93].

## 8. Alzheimer's Disease: A Neurodegenerative Disease

Proteostasis (tau and/or A*β* plaques) is a central component of pathology in Alzheimer' disease [94]. AD is characterised by the accumulation of intracellular neurofibrillary tangles, which are composed of microtubule binding protein tau assembled into paired helical and straight filaments [95] and the extracellular deposits of fibrillary A*β* [94]. Altered synaptic plasticity and synaptic loss correlates with cognitive dysfunction [96, 97]. Chronic intracerebral inflammation is also a pivotal factor driving neurodegeneration in the form of activated glia [98]. The amyloid hypothesis of Hardy and Selkoe [99] relies on intracerebral factors especially A*β* being pivotal to AD pathogenesis. Reactive oxygen/nitrogen species and the chronic activation of the complement cascade are all features of AD inflammatory pathology attributed to A*β* deposition [98].

The “aetiological” hypothesis accounts for extrinsic inflammatory factors contributing to the subclinical and clinical phases of the late-onset form of AD [6, 100–105]. Evidence from genome-wide studies suggests the innate immune system is involved in the onset of AD [106, 107] and this supports a role for microbes and/or their immunogenic components that classically initiate innate immune responses. Further evidence linking microbes to A*β* plaque deposition supports the ability of this hallmark protein to act as an antimicrobial peptide to counteract infections [108]. It is therefore not surprising that A*β* functions are related to innate immune defence mechanisms that mediate intrinsic responses [109]. In addition, multiple systemic infections can exacerbate premorbid cognitive status in AD patients and the current view indicates that this is the result of proinflammatory mediators crossing the BBB [93, 100, 101].

Poole et al. [6] reported that periodontal pathogen* P. gingivalis* components are also identified in AD subjects, and this could help to explain the associations of PD with the onset of, and perpetuating inflammation via, the keystone pathogen hypothesis described by Hajishengallis et al. [3]. In terms of the time-frame of the chronicity of inflammation of the two diseases under discussion, PD presents after 30 years of age [31], whereas the late-onset AD appears later (80+ years onwards) in life. Thus there is sufficient time for an established chronic periodontal pathogen such as* P. gingivalis* [110, 111] to exploit the haematogenous route [66] to access the brain as illustrated by Singhrao et al. [112].

Our recent work tested the proof of concept using ApoE^null^ mouse model of experimental periodontitis, whereby mice were periodontally infected with* P. gingivalis, T. denticola*,* T. forsythia,* and* Fusobacterium nucleatum* as mono- and polybacterial infections. Following chronic gingival infection and molecular identification using nucleotide sequencing, a statistically significant number of ApoE^null^ mice brains contained the* P. gingivalis *genomic DNA. These results demonstrated that the periodontal pathogen* P. gingivalis* was able to access the ApoE^null^ mice brains [7].

As this keystone pathogen remains in low amounts (10^3^) in the subgingival niche, the larger brain tissue ratio to number of actual* P. gingivalis* cells in the dementing human brain would make it difficult to detect by molecular methodologies. Given the resilience of* P. gingivalis* in the human host, even the presence of fewer* P. gingivalis* in the brain over at least three decades would be sufficient to contribute to a low but persistent level of local inflammation for its own nutritional sustenance and survival. One of the traits of a keystone inflammophilic pathogen is to establish communities of other microbes that can thrive under toxic inflammatory environment and contribute further to inflammation in the host. This could explain why specific microbes are also said to associate with AD hallmark protein A*β*. These include herpes simplex virus type I [104],* Chlamydophila pneumoniae* [103], and several species of spirochetes of which the well cited ones are* T. denticola* and* Borrelia burgdorferi* [91, 105]. Activation of the complement cascade in the AD brain can be direct via the hallmark proteins or from whole bacteria, or their components (LPS and peptidoglycan), and indirect activation via liberation of proinflammatory cytokines from other (TLR) signalling cascades [103–115]. Furthermore, liberation of reactive oxygen/nitrogen species also takes place when innate immune responses are activated by A*β* [98] and due to peptidoglycan from bacterial cell walls [40]. Such an environment is conducive to these inflammophilic pathogens in AD brains.

Our research also examined the inflammatory component arising from the innate immune responses relating to the dissemination of this bacterium into the brains of our ApoE^null^ mouse PD model. By using antibodies against the complement activation products of C3 convertase stage and the membrane attack complex on brain tissue sections from the ApoE^null^ mice, microglia in both infected and sham-infected groups, demonstrated strong intracellular labelling with C3 and C9. This was concluded as ongoing biosynthesis by microglial cells because Singhrao et al. [116, 117] had previously demonstrated that, in human brain tissue sections from another neurodegenerative condition, glia were the source of complement whilst the* in vitro* study [117] demonstrated that neurons were vulnerable from attack. Thus, from the infected mice brains, it was clear that pyramidal neurons of the hippocampus were opsonised with C3 activation fragments (*P* = 0.032) and were likely to have been vulnerable from activated complement [7]. The data from the human brains and that from the* in vivo* mouse study suggested specific associations of* P. gingivalis *with AD inflammatory pathology [7].

## 9. Implications of Inflammation in the Brain

Direct implications of bacterial infections in the elderly is said to affect memory [9, 100] via cytokine release in response to infection [100]. Indeed an increased amount of cytokines especially the macrophage secreted TNF-*α* has been reported in plasma of AD subjects [93]. Furthermore, elderly individuals appear to harbour a higher titre of circulating IgG from several periodontal pathogens [118], and clinical studies support this to correlate with a possible onset of mild cognitive impairment and even result in AD [119]. Persisting chronic peripheral/intrinsic inflammation is hypothesised to affect BBB capillary wall which likely prevents efflux of soluble/insoluble A*β* from the CNS. This hypothesis was tested following vaccination of anti-A*β* antibodies whereby amyloid plaque A*β* was shown to exit the brain via the cerebral blood vessel walls but failed and accessed the perivascular spaces instead [120].

## 10. An Overview of the Role of Periodontal Pathogens Modulating CNS Inflammation

Bacteria and/or their immunogenic components at the appropriate concentration initiate the classical innate immune signalling pathways via TLR-2 and TLR-4 mechanisms whereby the release of cytokines by microglia (IFN-*γ* and TNF-*α*) is an inevitable consequence. Chronic release of cytokines will eventually change the permeability at the BBB and reduce the efflux of A*β* from the CNS into the systemic circulation [121]. Under appropriate concentrations of LPS/peptidoglycan, TLR-2 and TLR-4 signalling, and release of reactive oxygen/nitrogen species such as superoxide ions, iNOS/NO, cytokine secretion and bacterial activation of the complement system become inseparable. Together these factors lead to vital neurons being destroyed and enhanced maintenance of chronic inflammation with consequences for development of disease [98, 113, 122–125]. Inheritable inflammatory traits related to cytokines exist within our population and especially in the decedents of those having suffered from the late-onset AD [126]. Thus, all immunogens that initiate innate immune responses of the host resulting in the liberation of proinflammatory cytokines may be detrimental in the individuals with inflammatory susceptibility traits.

## 11. Conclusions

Periodontal disease(s) are among the most common chronic infections of humans, characterized by the loss of periodontal ligament, connective tissue, and alveolar bone, and are a major cause of tooth loss. Major periodontal pathogens lead to chronic systemic inflammation due to recurrent transient bacteraemia, resulting in high levels of systemic cytokines and chemokines. Multiple epidemiological, clinical, and molecular studies have shown that PD associated chronic inflammation is associated with increased risk of dementia, including AD. Recently, an* in vivo* study in ApoE^null^ mice periodontally infected with* P. gingivalis* demonstrated the presence of* P. gingivalis* bacterial genomic DNA in the brain and activated the complement cascade [7]. The keystone hypothesis of Hajishengallis et al. [3] helps to explain the contribution that* P. gingivalis* may cause the* early* development of a neurodegenerative condition such as Alzheimer's disease. In our view,* P. gingivalis* (highly virulent strains) access the CNS during healthy stages but only those individuals with inflammatory susceptibility traits are likely to develop progressive inflammatory component representing neurodegenerative disease processes. Thus,* P. gingivalis* may be a missing link that, in time, will reveal if infection driven inflammation represents the early stage in the development of AD pathology followed by appearance of hallmark proteins.
